# Depressive Cognitions Impact On A Latent Growth Curve Model Of Depressive Symptoms In Caregiving Grandmothers

**DOI:** 10.1093/geroni/igab046.2905

**Published:** 2021-12-17

**Authors:** Christopher Burant, Carol Musil, Jaclene Zauszniewski, Alexandra Jeanblanc

**Affiliations:** Case Western Reserve University, Cleveland, Ohio, United States

## Abstract

Grandmothers caring for grandchildren have elevated levels of depressive symptoms compared to grandmothers who do not provide care. While the CES-D measures the somatic, positive and negative affect, and interpersonal strain symptoms experienced with depression, the Depressive CognitionScale © captures the change in cognitive thinking that often precedes depression. Depressive symptoms, on the other hand, are state like in nature and describe depressive symptoms that have happened recently. While depressive cognitions, according to Beck’s theory of depression, are the first negative thought processes to appear, these typically lead to other, more serious symptoms of depression. Specifically, depressive cognitions reflect negative thinking patterns and not depression. Data were collected on 343 participants in a longitudinal nationwide online research study of caregiving grandmothers. A latent growth curve model was used to track the trajectory of depressive symptoms at four time points (baseline, 2 weeks, 12 weeks, and 24 weeks). As depressive cognitions are the precursor to the development of depressive symptoms, a latent growth curve model was tested to gain an understanding of how depressive cognitions impacts the trajectory of depressive symptoms over time. The model fit the data well (Chi Square=21.025; df=9; p=.013; TLI=.976; CFI=.985; RMSEA=.063). Baseline depressive cognitions had a strong impact on the intercept (Standardized Beta=.76, p<.001) and the slope of depressive symptoms (Standardized Beta=-.67, p<.001). The continued impact of depressive cognitions over 24 weeks indicates the need for potential interventions to further address depressive cognitions as a way to decrease depressive symptoms in grandmother caregivers.

